# Heterogeneity in macrophages along the cochlear spiral in mice: insights from SEM and functional analyses

**DOI:** 10.3389/fncel.2023.1222074

**Published:** 2023-08-25

**Authors:** Celia Zhang, Mengxiao Ye, Peter Bush, Bo Hua Hu

**Affiliations:** ^1^Department of Communicative Disorders and Sciences, University at Buffalo, Buffalo, NY, United States; ^2^Department of Audiology, University of the Pacific, San Francisco, CA, United States; ^3^South Campus Instrument Center, University at Buffalo, Buffalo, NY, United States

**Keywords:** macrophage, cochlea, morphology, scanning electron microscopy (SEM), aging, noise

## Abstract

The susceptibility of sensory cells to pathological conditions differs between the apical and basal regions of the cochlea, and the cochlear immune system may contribute to this location-dependent variability. Our previous study found morphological differences in basilar membrane macrophages between the apical and basal regions of the cochlea. However, the details of this site-dependent difference and its underlying structural and biological basis are not fully understood. In this study, we utilized scanning electron microscopy to examine the ultrastructure of macrophages and their surrounding supporting structures. Additionally, we examined the phagocytic activities of macrophages and the expression of immune molecules in both apical and basal regions of the cochlea. We employed two mouse strains (C57BL/6J and B6.129P-*Cx3cr1^*tm*1*Litt*^/*J) and evaluated three experimental conditions: young normal (1–4 months), aging (11–19 months), and noise-induced damage (120 dB SPL for 1 h). Using scanning electron microscopy, we revealed location-specific differences in basilar membrane macrophage morphology and surface texture, architecture in mesothelial cell layers, and spatial correlation between macrophages and mesothelial cells in both young and older mice. Observations of macrophage phagocytic activities demonstrated that basal macrophages exhibited greater phagocytic activities in aging and noise-damaged ears. Furthermore, we identified differences in the expression of immune molecules between the apical and basal cochlear tissues of young mice. Finally, our study demonstrated that as the cochlea ages, macrophages in the apical and basal regions undergo a transformation in their morphologies, with apical macrophages acquiring certain basal macrophage features and vice versa. Overall, our findings demonstrate apical and basal differences in macrophage phenotypes and functionality, which are related to distinct immune and structural differences in the macrophage surrounding tissues.

## 1. Introduction

The cochlea is the sensory organ for hearing, and its sensory cells are vulnerable to various pathological processes such as acoustic injury, ototoxicity, and aging. Research indicates that hearing loss typically begins in higher frequencies and then advances toward the low- and mid-frequencies (Gates et al., 1990; Tarter and Robins, 1990; Fausti et al., 1992; Wilcox et al., 2000). This pathogenesis pattern corresponds to the progression pattern of sensory cell lesions, which typically starts at the basal end of the cochlea, and then spreads toward the apical region. While the underlying mechanisms responsible for this phenomenon are not fully understood, evidence suggests that the cochlear immune system is critical for controlling the sensory cell microenvironment and affecting their survival and pathogenesis (Hirose et al., 2005; Tan et al., 2013; Fujioka et al., 2014; Okano, 2014; Hu et al., 2018).

Our research has demonstrated that more than 80% of known immune-related genes are constitutively expressed in the cochlea under normal conditions, and many of these genes become overexpressed following cochlear stress (Patel et al., 2013; Cai et al., 2014; Yang et al., 2016). Inflammatory molecules have been implicated in various cochlear pathogeneses (Satoh et al., 2002; Cho et al., 2004; Miyao et al., 2008; Han et al., 2012). Moreover, disrupting the expression of immune-related genes has been shown to alter cochlear responses to stress (Sautter et al., 2006; Calton et al., 2014). These findings highlight the importance of the cochlear immune system in maintaining sensory cell homeostasis and contributing to its pathogenesis. However, it is still uncertain whether there are differences in the immune environment between the apical and basal cochlear regions.

Macrophages are present in the cochlea. These cells are involved in immune surveillance and play a critical role in preserving the functional integrity of cochlear structures including the blood-perilymph barrier, inner hair cell synapses, and ganglion neurons (Kaur et al., 2017; Hirose and Li, 2019; Manickam et al., 2023). In response to pathological insults, circulating monocytes migrate from microvessels to the cochlea and participate in the cochlear inflammatory response to tissue damage. They produce various inflammatory molecules (Tornabene et al., 2006; Okano et al., 2008; Frye et al., 2018; Hu et al., 2018; Zhang et al., 2020) and are responsible for the phagocytosis of damaged cells (Fredelius, 1988; Fredelius and Rask-Andersen, 1990; Hirose et al., 2017). While there is compelling evidence connecting cochlear macrophages to the survival of sensory cells, ganglion cells, and hair cell synapses, the reported effects can vary under different experimental conditions (Kaur et al., 2017; Manickam et al., 2023; Zhang et al., 2023). For example, Manickam et al. (2023) showed the protective function of cochlear macrophages in inner hair cell ribbon synapses during acoustic injury. Conversely, macrophages have also been implicated in detrimental roles in cochlear pathogenesis, such as neomycin-induced ototoxicity and genetic mitochondrial dysfunction in the cochlea (Sun et al., 2015; Zhang et al., 2023).

Cochlear macrophages are distributed in various regions of the cochlea in both humans and rodents (Hirose et al., 2005; Shi, 2010; Liu et al., 2018), including the spiral ligament, stria vascularis, basilar membrane, osseous spiral lamina, spiral limbus, and modiolus. However, the organ of Corti, where sensory cells reside, lacks immune cells under normal conditions (Hirose et al., 2005; Lang et al., 2006; Okano et al., 2008; Sato et al., 2008; Du et al., 2011). The macrophage population closest to the organ of Corti is the basilar membrane macrophages, which are located on the scala tympani side of the basilar membrane. Since sensory cells in the organ of Corti are particularly vulnerable to cochlear pathogenesis, studying basilar membrane macrophages can help with understanding the underlying mechanisms of sensory cell pathogenesis, including the apical and basal differences in sensory cell vulnerability to stress.

Macrophages are remarkably adaptable immune cells capable of assuming diverse morphologies and functions in response to their microenvironmental cues (Wynn et al., 2013). In cases of acute stress, circular monocytes infiltrate the cochlea and gradually transform into macrophages with distinct morphologies and functional characteristics. These macrophages undergo alterations such as increased body size, process formation, and upregulation of inflammatory molecule expression. Our previous research has demonstrated that the morphology of basilar membrane macrophages is location-dependent (Yang et al., 2015; Frye et al., 2017; Zhang et al., 2020). Specifically, macrophages in the apical region of the cochlea display a dendritic shape with numerous branches and fine processes, while those in the middle region exhibit trunk-like or branched morphologies. In contrast, macrophages in the basal region of the cochlea display a more rounded or globular shape. Further studies have revealed that these unique location-dependent morphological characteristics emerge as the cochlea undergoes postnatal maturation (Dong et al., 2018). Currently, it is unknown whether basilar membrane macrophages with different morphologies differ in their function and what biological factors contribute to the topographical differences.

The objectives of our study were to: (1) use scanning electron microscopy to reveal ultrastructural details of macrophages; (2) investigate the phagocytic activity of macrophages; and (3) identify potential factors that contribute to the observed differences in macrophage morphology between the apical and basal regions. We selected C57BL/6J mice for our study due to their extensive utilization in the field of hearing sciences. C57BL/6J mice display an early onset of hearing loss and sensory cell pathogenesis, allowing us to study the effects of aging on macrophage activities. We also used the B6.129P-*Cx3cr1^*tm*1*Litt*^*/J mice for our study. These mice carry the enhanced green fluorescent protein (EGFP) sequence replacing the first 390 bp of the coding exon (exon 2) of the chemokine (C-X3-C motif) receptor 1 (*Cx3cr1*) gene. This strain was selected due to the green fluorescence exhibited by its macrophages, which facilitates their identification during *ex vivo* experiments. Results from scanning electron microscopy revealed that macrophages on the basilar membrane exhibit significant differences in morphology, surface texture, and interaction with mesothelial cells along the cochlear spiral. Furthermore, we found that these cells differed not only in their morphologies but also in their phagocytic activity, with basal macrophages displaying greater phagocytic activity. Further analyses of the macrophage environment revealed differences in both the expression levels of proinflammatory genes and the architecture of the surrounding mesothelial cell layer. These variations may contribute to the location-dependent differences in macrophage morphology. Lastly, our study revealed that during the aging process, apical macrophages acquire basal macrophage characteristics and vice versa. Collectively, our results support the existence of distinct macrophage phenotypes and functions in the apical and basal regions of the cochlea, which are likely influenced by variations in the immune and structural properties of the surrounding tissues.

## 2. Materials and methods

### 2.1. Animals

Both male and female C57BL/6J mice between the ages of 1 and 19 months (Jackson Laboratory, Bar Harbor, ME, USA) were used in this investigation. B6.129P-*Cx3cr1^*tm*1*Litt*^*/J mice that have an enhanced green fluorescent protein (EGFP) sequence replacing the first 390 bp of the coding exon (exon 2) of the chemokine (C-X3-C motif) receptor 1 (*Cx3cr1*) gene were also used (Jung et al., 2000). This strain was chosen because its macrophages exhibit green fluorescence, which aids in identifying macrophages during *ex vivo* experiments.

The mice were housed at the Laboratory Animal Facility of the University at Buffalo and were shielded from excessive noise during the entire investigation. To minimize the use of animals while ensuring an adequate sample size, both cochleae of each mouse were harvested and allocated to different experiments. The number, age, and strain of mice utilized in each experimental condition are summarized in Table 1. All protocols pertaining to the treatment and handling of the animals were sanctioned by the Institutional Animal Care and Use Committee of the State University of New York at Buffalo.

**TABLE 1:** Number of ears used in each experimental condition.

Conditions		# of ears	Strain	Age
SEM observation	Macrophages in normal ears	7	C57BL/6J	1–4 months
	Mesothelial cells in normal ears	7	C57BL/6J	1–4 months
	Macrophages for phagocytosis in noise-damaged ears	10	C57BL/6J	4–6 weeks
	Macrophages in aging ears	9	C57BL/6J	11–19 months
Immunohistochemistry for macrophages	Noise-damaged ears	4	C57BL/6J	4–6 weeks
	Normal ears	4	C57BL/6J	4–6 weeks
*Ex vivo* culture with latex beads for macrophage phagocytosis	Noise-damaged ears	2	B6.129P-*Cx3cr1^*tm*1*Litt*^*/J	4–6 weeks
	Aging ears	2	B6.129P-*Cx3cr1^*tm*1*Litt*^*/J	11 months
	Control ears	2	B6.129P-*Cx3cr1^*tm*1*Litt*^*/J	4–6 weeks
Expression analysis of immune molecules	Control ears	5	C57BL/6J	4–6 weeks
Hair cell quantification	Young ears	6	C57BL/6J	4–6 weeks
	Aging ears (1)	6	C57BL/6J	11–12 months
	Aging ears (2)	6	C57BL/6J	17 months

### 2.2. Acoustic overstimulation

Acoustic overstimulation was used to induce cochlear damage for assessing macrophage activities under stress. The animals were subjected to a continuous noise of 1–7 kHz at 120 dB SPL for 1 h. This noise condition was chosen because it is known to cause permanent hearing loss and sensory cell death (Zhang et al., 2020), enabling us to investigate the cochlear immune response in the context of hair cell pathogenesis. The noise was produced utilizing a Real-Time signal processor (RP2.1, Tucker Davis Technologies, TDT, Alachua, FL, USA) and was directed through an attenuator (PA5, Tucker Davis Technologies, Alachua, FL, USA), a power amplifier (Crown XLS 202, Harman International Company, Elkhart, IN, USA), and a loudspeaker (NSD2005-8, Eminence, Eminence, KY, USA) positioned 30 cm above the animal’s head. The sound level at the animal’s head position in the sound field was assessed with a sound level meter (LD-PCB, model 800 B, APCB Piezotronics Div., Larson Davis, Depew, NY, USA), a preamplifier (LD-PCB, model 825, Larson Davis, Depew, NY, USA), and a condenser microphone (LDL 2559, Larson and Davis, Depew, NY, USA). The mice were placed individually in a cylindrical holding cage with an approximate diameter of 5.5 cm and a height of 6.5 cm during the noise exposure.

### 2.3. Sample collection

Upon completion of the experimental treatment or at a defined age (refer to the section “3. Results” for specific details), the mice were euthanized under CO_2_ anesthesia by decapitation. The cochleae were promptly extracted from the skull and prepared for the following analyses.

### 2.4. Scanning electronic microscopy (SEM)

Scanning electron microscopy (SEM) was used to reveal the surface structures of the scala tympani with a particular focus on macrophages and mesothelial cells on the basilar membrane. The cochleae were fixed with 2% glutaraldehyde in 10 mM phosphate-buffered saline (PBS) at room temperature for 1 h followed by refrigeration at 4°C for at least overnight. The cochleae were then decalcified with 10% ethylenediaminetetraacetic acid (EDTA) at 4°C for 5 days and dissected to expose the luminal surface of the scala tympani, which included the sensory epithelium, lower section of the spiral ligament, and osseous spiral lamina of the entire cochlea, as well as the lateral wall of the scala tympani at the basal turn of the cochlea. The tissues were dehydrated in a graded series of ethanol (30, 50, 70, 85, 95, and 100%) for 15 min each and then with 100% hexamethyldisilazane for 2 h. The tissues were then mounted onto tissue stages and coated twice, first with evaporated carbon and then with gold, using a high vacuum evaporator (Denton 502 Evaporator, Denton Vacuum, LLC, Moorestown, NJ, USA). The tissues were examined and photographed using a field emission SEM (Hitachi SU-70, Tokyo, Japan) at 2.0 keV using an in-lens secondary electron detector at zero tilt, or a lower detector at 70° tilt. Cochleae with varying experimental conditions were examined (see Table 1), and several hundred SEM images were collected (refer to the section “3. Results” for the sample size of each experimental condition). To improve image contrast and highlight specific cells, we utilized Adobe Photoshop CS6 to color the cells of interest in the images presented in this paper.

### 2.5. Immunohistochemistry and confocal microscopy

Immunohistochemistry was performed to observe immune cells for the macrophage phagocytosis experiment. Cochleae were fixed with 10% buffered formalin and then decalcified using 10% EDTA at 4°C for 5 days. The cochleae were dissected in 10 mM PBS to collect whole-mount tissues containing the sensory epithelium, lower section of the spiral ligament, and osseous spiral lamina. Following dissection, whole-mount preparations were treated with 0.5% Triton X-100 to permeabilize the cells for 30 min at room temperature and then incubated in a blocking buffer (pH 7.4) for 1 h at room temperature. The tissues were subsequently incubated overnight at 4°C with an anti-mouse CD45 (goat polyclonal IgG, AF114, R&D System) at a concentration of 1:100 in 0.5% BSA and 0.25% Triton X-100 buffer. After incubation, the tissues were rinsed three times with 10 mM PBS and then incubated with a secondary antibody (Alexa Fluor 568 donkey anti-goat IgG, A11057, Thermo Fisher) at a concentration of 1:100 in 0.5% BSA for 2 h. The tissues were further stained with propidium iodide (50 μg/ml in 10 mM PBS) for 10 min. Following three rinses with 10 mM PBS, the tissues were mounted onto slides and examined using a Zeiss ELYRA confocal microscope.

### 2.6. *Ex vivo* culture with latex beads

To assess the phagocytic activities of cochlear macrophages, we performed an *ex vivo* observation of macrophage phagocytosis of latex beads (Fluoresbrite Polychromatic Red Microspheres, 0.5 μm, Polysciences, Inc., Warrington, PA, USA). Upon euthanasia of animals, the cochleae were quickly removed, and both round and oval windows were opened. Through the round window, the cochlea was slowly perfused with a freshly prepared latex bead suspension. Specifically, the stock solution was diluted (1:30) with a macrophage culture medium (RPMI 1640, Life Technologies, Grand Island, NY, USA), supplemented with 10% FBS (Atlanta Biologicals, Flowery Branch, GA, USA), 2 mM L-glutamine, and 1% penicillin-streptomycin-neomycin solution (Life Technologies, Grand Island, NY, USA). The cochleae were incubated at 37°C for 20 to 40 min. After incubation, the cochleae were perfused with cold 10 mM PBS twice to wash out excessive latex beads and then fixed with 10% formalin overnight. The cochleae were dissected in 10 mM PBS and the sensory epithelia, osseous spiral lamina, and the lower portion of the spiral ligament were collected. The collected tissues were mounted on slides and examined using a confocal microscope.

### 2.7. Assessment of sensory cell damage

The cochleae were fixed overnight with 10% buffered formalin at 4°C and were dissected in 10 mM PBS to expose the sensory epithelia. To observe the nuclei of hair cells in the sensory epithelia, the tissues were stained with TO-PRO-3 (1 μM in 10 mM PBS) for 30 min or 4′,6-diamidine-2′phenylindole dihydrochloride (DAPI) (1 mg/ml in PBS) for 10 min. After the staining, tissues were rinsed three times in 10 mM PBS. Then the tissues were imaged using a fluorescence microscope (Leica Z6 APO Manual MacroFluo, 10× objective) equipped with a Leica DFC digital camera. Using Adobe Photoshop CS6, sections of individual images were aligned and stitched together to generate a panoramic view of the entire cochlear spiral. Sensory cells were quantified at 150 μm intervals from the apex to the base along the cochlear spiral.

### 2.8. Real-time quantitative polymerase chain reaction (RT-qPCR)

Real-time quantitative polymerase chain reaction was performed to determine the transcriptional expression of the following genes: *Tnf*, *Il-1ß*, *Ccl2*, *Ccl3*, and *Ccl4*. Animals were sacrificed, and the cochleae were quickly collected. Following dissection, cochleae were placed in an RNA stabilization reagent (RNAlater, Qiagen, Valencia, CA, USA) to collect the sensory epithelium and lateral wall. The collected tissues were transferred to RNase-free PCR tubes and stored at −80°C before RNA extraction. Tissue from one cochlea was used to generate one sample. Total RNA was extracted using the RNeasy Micro Kit (Catalog #74034, Qiagen, Valencia, CA, USA) as per manufacturer’s instruction.

The concentration and quality of isolated total RNAs were assessed using the NanoDrop 1000 (Thermo Fisher Scientific, Waltham, MA, USA). The isolated total RNAs were reverse transcribed into cDNA using a High Capacity cDNA Reverse Transcription Kit (SuperScript^TM^ VILOTM MasterMix, Invitrogen, Carlsbad, CA, USA). The transcriptional expression levels of the target genes were examined using pre-developed TaqMan gene expression primer/probe assays (Applied Biosystems, Foster City, CA, USA). Synthesized cDNA was mixed with a target gene probe and a TaqMan Universal PCR Master Mix (Thermo Fisher Scientific, Waltham, MA, USA) and transferred to a 96-well plate. RT-qPCR was performed in a CFX Connect Real-Time PCR detection system (Bio-Rad, Hercules, CA, USA). Pre-developed GAPDH and RPL13A gene expression assays (Applied Biosystems, Foster City, CA, USA) were used as endogenous controls. Analysis of relative gene expression data was completed using a previously reported standard 2^–ΔΔ*Ct*^ method (Livak and Schmittgen, 2001).

### 2.9. Quantitative analyses of basilar membrane macrophages

Macrophage phenotype and function was evaluated and described per the following criteria. ***Length of cell processes*:** One representative macrophage was selected from each cochlea and three macrophage processes from each cell were measured in young and aging mice (*n* = 6 cochleae per group). The measurement was conducted by tracing the length of macrophage processes, which represent the characteristic profile of the macrophage, using Adobe Photoshop CS6. The lengths of 18 macrophage processes for each group were then averaged to provide a single representative number for young and aging mice. ***Macrophage uptake of latex beads*:** The ability of macrophages to uptake latex beads was examined to define their phagocytic function. Since individual latex beads were difficult to quantify, we examined the mean gray value of the latex beads within a single macrophage. This was achieved using Adobe Photoshop CS6 to trace the cell boundary of each cell. The *record observations* function of the software provided a measurement of the gray value, which represents the level of latex bead accumulation in the cell. The gray value of 18 macrophages for each group (*n* = 6 cochleae per group) were averaged to provide a single representative value for the intensity of accumulated latex beads in the cell.

### 2.10. Statistical analyses

All data are presented as mean ± 1 standard deviation. Statistical analyses were performed using SigmaStat (version 10.0.1.25, San Jose, CA, USA) or GraphPad Prism (Version 9.3.1). Group means were statistically compared using a two-tailed Student’s *t*-test. An α-level of 0.05 was chosen to denote significance for all statistical tests. Normative data and equal variance tests were performed for all statistical analyses. If these two criteria were not satisfied, non-parametric tests were performed.

## 3. Results

**FIGURE 1:**
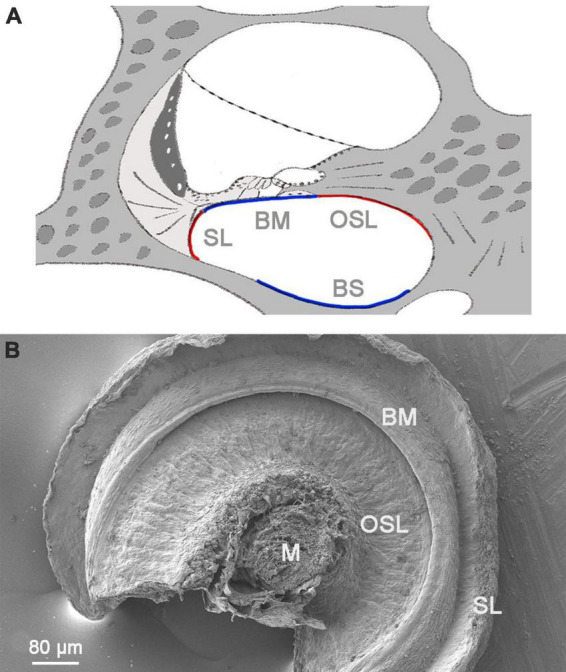
A low-magnification view of the cochlear structure for SEM. **(A)** A schematic illustrating a side view of the cochlea. The red and blue lines highlight the regions of the scala tympani that were observed using SEM. **(B)** An SEM view of the scala tympani side of the basilar membrane, spiral ligament, and osseous spiral lamina. The image captures the region spanning approximately 30–70% of the cochlear spiral. M, modiolus; OSL, osseous spiral lamina; BM, basilar membrane; SL, spiral ligament; BS, bony shell.

We utilized a modified whole-mount preparation technique to prepare cochlear tissues for SEM imaging. This approach facilitated our observation of macrophages present on the scala tympani side of the basilar membrane, osseous spiral lamina, and the lower portion of the spiral ligament, as well as the luminal surface of the scala tympani against the cochlear bony shell (Figure 1).

Macrophages were identified based on their distinctive shape, size, surface texture, and location. These cells were easily distinguishable in the middle and basal regions of the cochlear spiral, where they displayed typical macrophage morphologies. However, in the apical section of the cochlea, some macrophages are similar in shape and size to adjacent mesothelial cells, making differentiation challenging. To avoid any potential confusion, we excluded cells whose identity we were uncertain about from the subsequent analyses. By comparing the SEM data with our previous observations of cochlear macrophages through immunostaining, we estimated that the macrophages analyzed using SEM represent approximately 50–60% of the total macrophage population in the respective region. Although this approach might have resulted in an underrepresentation of apical macrophages, it increased our certainty in the accuracy of macrophage identification. As a result, our analysis focused on characterizing the morphological features of macrophages rather than quantifying their numbers. This approach allowed us to gain a more detailed understanding of the structural characteristics of macrophages and their interactions with the surrounding tissues.

### 3.1. Basilar membrane macrophages exhibit location-specific morphology along the cochlear spiral

Our previous immunohistochemistry studies demonstrated that cochlear macrophages display a range of shapes and sizes along the longitudinal axis of the cochlea (Yang et al., 2015; Frye et al., 2017). The present SEM observations provide further details on this diversity by revealing the ultrastructural characteristics of macrophages. We categorized macrophages on the luminal surface of the scala tympani into four groups based on their locations (Figure 1): those on the surface of the basilar membrane, referred to as basilar membrane macrophages, those on the surface of the osseous spiral lamina, referred to as osseous spiral lamina macrophages, those on the surface of the lower portion of the spiral ligament, referred to as spiral ligament macrophages, and those on the remaining luminal surface of the scala tympani against the bony shell of the cochlea, referred to as bony shell macrophages. The analysis of macrophage morphology presented here was based on SEM examination of seven cochleae obtained from both male and female C57BL/6J mice aged 1 to 4 months, which served as representative samples for normal conditions.

#### 3.1.1. Basilar membrane macrophages

**FIGURE 2:**
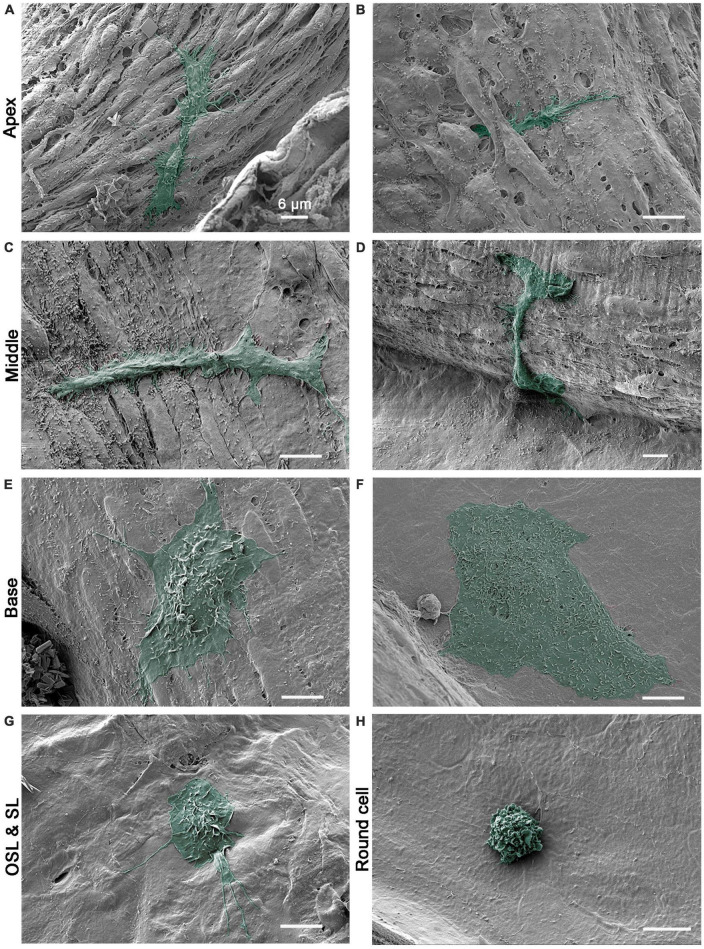
Scanning electronic microscopy (SEM) images of macrophages with diverse morphologies along the cochlear spiral. **(A,B)** Typical morphology of apical macrophages that reside on the basilar membrane at the apical 30% of the cochlear spiral. The macrophage has an elongated body and fine processes that are firmly attached to surrounding mesothelial cells. In panel **(B)**, the macrophage can be observed with its body and processes extending beneath the neighboring mesothelial cells. **(C,D)** Typical morphology of basilar membrane macrophages in the middle region of the cochlear spiral (30–60% from the apex). The macrophages have a trunk-like morphology with short processes. The surface of the cell body is relatively smooth. **(E,F)** Typical morphology of basilar membrane macrophages in the basal 40% of the cochlear spiral. One macrophage exhibits an amoeboid shape with short lamellipodia at the edge and small ruffles and pits on its top. The other cell has a large flat body with an irregular shape and microvilli on the surface. **(G)** Typical morphology of macrophages on the surface of osseous spiral lamina. The cell has a rounded shape with lamellipodia and fine processes. **(H)** A small round cell with multiple ruffles on the surface, which is a morphological feature of the monocyte. Scale bars in all panels = 6 μm.

Basilar membrane macrophages reside close to outer hair cells, a vulnerable cell type in the cochlea. Macrophages in this region displayed a site-specific morphology. In the apical portion of the cochlea (i.e., the apical 30% of the cochlear spiral), macrophages displayed spindle-like or elongated bodies with delicate processes that extend to contact neighboring mesothelial cells (Figure 2A). Their surface lacked ruffles but had bumps and tips. While many macrophages were observed on the surface of the basilar membrane, positioned on top of the mesothelial cells, others had their bodies or processes that extended beneath mesothelial cells (Figure 2B), bringing them closer to the organ of Corti.

In the middle section of the cochlea (approximately 30–60% of the cochlear spiral), macrophages exhibited a trunk-like or branched shape. Unlike the apical macrophages, they did not have elongated and slender processes, and their surface was generally smooth with some bumps or small folds (Figures 2C, D).

In the basal section of the cochlea (approximately the basal 40%), macrophages were more globular with short processes that extended to nearby mesothelial cells (Figure 2E). Their bodies were often bulky, with flat edges and numerous small ruffles, folds, and tips on the surface. Occasionally, flat cells with a large body were visible (Figure 2F). These cells had many small pits on their surface. Unlike apical macrophages, macrophages located in the middle and basal sections typically had cell bodies and processes covering the surface of surrounding cells, without extensions beneath them.

#### 3.1.2. Osseous spiral lamina and spiral ligament macrophages

Macrophages on the surface of the osseous spiral lamina, spiral ligament, and other regions of the inner surface of the scala tympani displayed diverse morphologies. These cells often had a stingray morphology or an irregular shape with surface folds and a few long, thin processes (Figure 2G). Unlike basilar membrane macrophages, these cells did not exhibit a clear apical-basal distinction in their morphology.

In addition to macrophages with large bodies, we also observed a few immune cells with a small round shape per cochlea. These cells were not confined to a specific location and were found in all areas we examined. Most small round cells exhibited a monocyte-like morphology with surface ruffles, as seen in Figure 2H. Our previous studies using immunohistology have shown that small round cells express monocyte markers, including Iba1 and Ly6C (Frye et al., 2017; Zhang et al., 2020). Collectively, our SEM observations were consistent with our previous immunohistological analyses of cochlear macrophages and provided additional details about macrophage surface textures at the ultrastructural scale. These observations confirmed the presence of an apical-to-basal difference in macrophage morphology.

### 3.2. Phagocytic activities in macrophages with different morphologies

To explore potential functional differences among macrophages with distinct morphologies, we assessed their phagocytic activity within the cochlea in the presence of cell debris. To simulate a model of cochlear pathogenesis, we utilized acoustic overstimulation, as this damaging factor induces acute sensory cell damage and promote robust macrophage activities (Fredelius et al., 1988; Fredelius and Rask-Andersen, 1990; Hirose et al., 2005; Tornabene et al., 2006; Wakabayashi et al., 2010). Mice were exposed to broadband noise at 120 dB SPL for 1 h. For the following SEM analyses, we examined a total of 10 cochleae collected 2 to 20 days after noise exposure from male and female C57BL/6J mice aged 4–6 weeks. Age-matched young C57BL/6J mice of both sexes, which were not subjected to acoustic injury, were used as normal controls.

**FIGURE 3:**
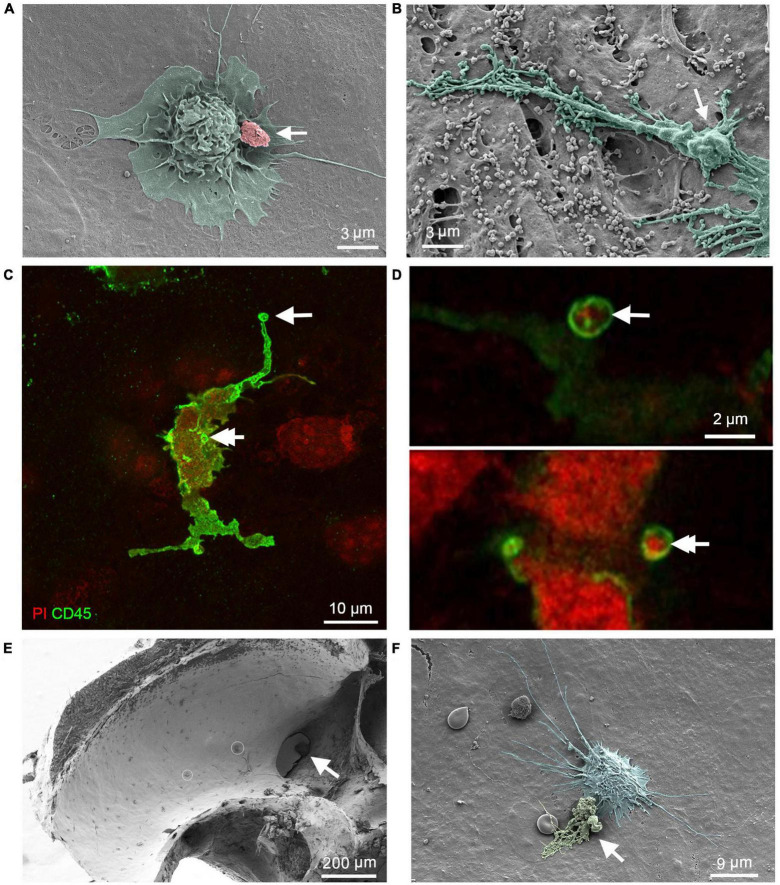
Macrophage engulfment of cell debris. **(A)** SEM image showing a basilar membrane macrophage after exposure to a broadband noise of 120 dB SPL for 1 h. Cell debris can be seen on the surface of the macrophage (arrow), which could correspond with the initial step of phagocytosis. **(B)** SEM image showing the granular appearance (arrow) of a macrophage process, suggesting that it may have engulfed debris. **(C,D)** Confocal microscopy images show a macrophage stained with an antibody against CD45, a pan-immune cell marker protein, and propidium iodide, a nuclear probe. The cochlea was examined at 6 days after noise exposure of 120 dB SPL for 1 h. Arrows point to nuclear fragments within the macrophage processes, suggesting macrophage engulfment of nuclear fragments [see **(D)** for magnified views of the processes indicated by the two arrows in **(C)**]. **(E)** SEM image showing the accumulation of macrophages and cell debris on the lateral wall of the scala tympani near the opening of the cochlear aqueduct (arrow) 6 days after exposure to an intense noise of 120 dB SPL for 1 h. The small circles mark cell debris. **(F)** An enlarged view of a macrophage with a rounded shape and many long projects in the lateral wall of the scala tympani. The arrow indicates cell debris in contact with the macrophage.

We found cell debris attached to the surface of macrophages (Figure 3A), which could correspond with the initial step of engulfment. In addition, we noted that macrophage processes exhibited a granular appearance, indicating the presence of granules within the processes (Figure 3B). To provide evidence that surface granules indicate engulfment of cell debris, we used confocal microscopy to examine two cochleae from two mice collected at 6 days after the noise exposure. The tissues were stained with an immune cell marker protein, CD45, for immune cell identification and with propidium iodide for nuclei. Figures 3C, D show nuclear fragments within the processes of a macrophage, which is an indication of macrophage engulfment of nuclear fragments. We also examined the luminal surface of the scala tympani. Pieces of cell debris were more frequently seen in the basal end of the scala tympani, particularly in the regions close to the opening of the cochlear aqueduct (Figure 3E). In this region, cell debris were often observed in direct contact with macrophages (Figure 3F). Such debris attachment to macrophages were not observed in normal control ears (see Figure 2).

To explore potential functional differences between macrophages in the apical and the basal region, we performed *ex vivo* observations of macrophage phagocytosis by measuring their ability to uptake latex beads. This analysis allowed us to quantify the phagocytic capabilities of individual macrophages and assess potential functional differences between the apical and basal macrophages on the basilar membrane. We utilized B6.129P-*Cx3cr1^*tm*1*Litt*^*/J mice of both sexes for our study, as their macrophages exhibit green fluorescence, aiding in the identification of macrophages during analysis. Macrophages were examined under two stress conditions: acoustic injury and aging, in order to broaden the scope of our analysis. Since no noticeable differences were observed between these two conditions, data from observations of acoustic injury (1-day post noise exposure, *n* = 2) and aging (10 months, *n* = 2) were combined to analyze the differences between apical and basal macrophages. The cochleae from young mice (*n* = 2) without acoustic overstimulation were also examined and the data were not included in the statistical analysis.

**FIGURE 4:**
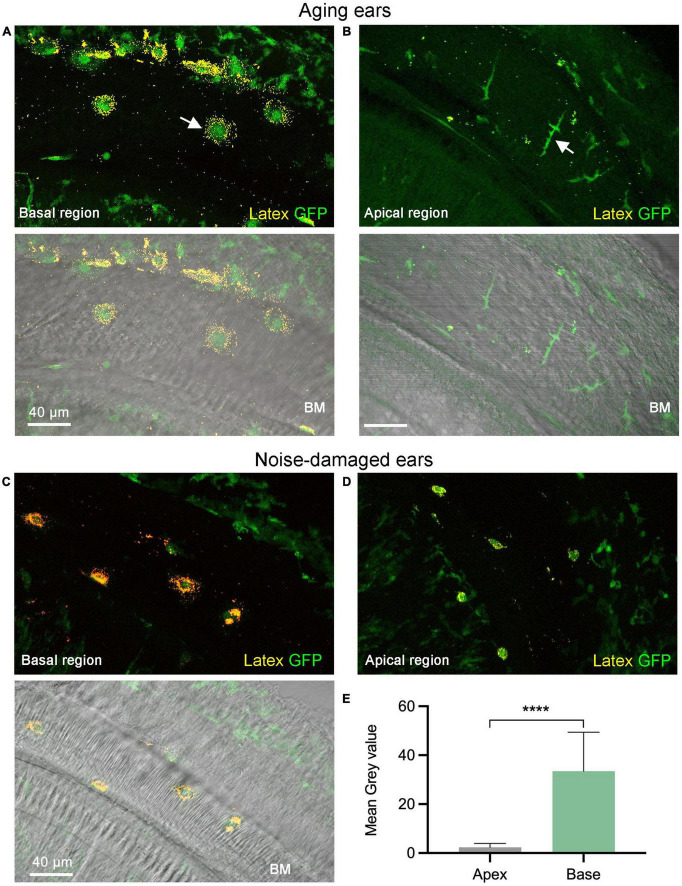
Comparison of phagocytic activity between the apical and basal macrophages. **(A,B)** Accumulation of latex beads in macrophages of an aging cochlea at 11 months of age. Notice that the basal macrophages exhibit a higher number of latex beads compared to the apical macrophages (pointed by the arrow). The lower two panels in both **(A,B)** feature differential interference contrast views of the tissues, which offer information regarding tissue orientation. Please note that the macrophages in the upper-right corner and bottom-left corner correspond to cells located within the lateral wall and the osseous spiral lamina, respectively. These cells do not have latex beads because they lack direct contact with the latex beads. **(C,D)** Accumulation of latex beads in macrophages examined 1-day post-exposure to a broadband noise of 120 dB SPL for 1 h. Notice that basal macrophages display more latex beads than apical macrophages. **(E)** A comparison of the mean gray level of the latex bead fluorescence reveals a significantly stronger latex bead fluorescence intensity for the basal macrophages than the apical macrophages (*n* = 18 macrophages each group which contains 4 cochleae with 2 cochleae from aging mice and 2 cochleae from noise-damaged mice). BM, basilar membrane; GFP, green fluorescence protein from B6.129P-*Cx3cr1^*tm*1*Litt*^*/J mice that marks macrophages. ****P < 0.0001; two-tailed unpaired Student’s *t*-test.

In all examined cochleae, basal macrophages displayed more accumulation of microbeads compared to apical macrophages (Figures 4A–D). Further quantitative analysis of the mean gray value associated with latex beads confirmed this observation (two-tailed unpaired student’s *t*-test, *P* < 0.0001) (Figure 4E). This analysis revealed that macrophages in the basal region of the cochlea exhibit stronger phagocytic activity than those in the apical region of the cochlea. In the control cochlea, macrophages exhibited a comparable difference between the apical and basal regions. However, it was observed that macrophages had significantly fewer latex beads.

### 3.3. Location-dependent differences in the architectural features of the mesothelial cell layer

To determine the factors that may contribute to the development of location-specific morphologies in basilar membrane macrophages, we examined the macrophage environment. Mesothelial cells, which line the scala tympani side of the basilar membrane, provide structural support to macrophages. Our aim was to investigate whether this macrophage-supporting layer exhibits a location-dependent difference in its architectural framework. To achieve this, we used SEM to examine the ears of young mice of both sexes (1–4 months, *n* = 7 cochleae), which served as representative samples for normal conditions.

**FIGURE 5:**
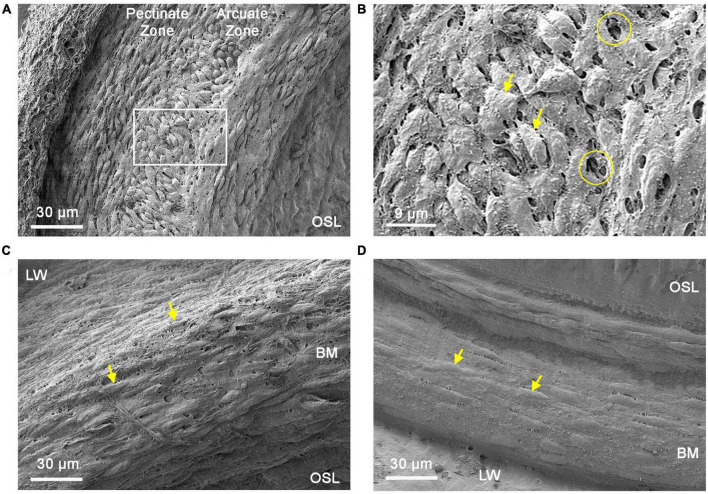
Comparison of mesothelial cell layer morphologies between the apical and basal cochlear spiral. **(A)** Typical morphology of the mesothelial cell layer in the apical region of the cochlear spiral (apical 30% of the cochlear spiral). Mesothelial cells in this region are spindle-shaped and densely packed. **(B)** Enlarged view of the region marked by the square in panel **(A)**. The circles mark saccular spaces between mesothelial cells and arrows indicate mesothelial cells. Arrows point to mesothelial cells. **(C)** Typical morphology of the mesothelial cell layer in the transition region (30–40% from the apex) toward the middle and basal portions of the cochlear spiral. In this region, mesothelial cells have a fusiform shape and flatter cell bodies (arrows). Tunnel-like spaces between mesothelial cells are less prominent compared to those observed in the apical portion of the cochlear spiral. **(D)** Typical morphology of the mesothelial cell layer in the middle and basal portion (50–70% from the apex) of the cochlear spiral. These cells are relatively flat and loosely packed (arrows). In contrast to the apical region, there are few saccular spaces between the cells in this area. BM, basilar membrane; OSL, osseous spiral lamina; LW, lateral wall.

Figures 5A, B display surface views of mesothelial cells located in the apical region of the cochlea. In this region, mesothelial cells were bulky and densely packed, forming a continuous membranous layer over the basilar membrane. The orientation of these cells varied based on their location, with cells in the lateral region corresponding to the pectinate zone situated longitudinally, while those in the medial portion corresponding to the arcuata zone became disoriented. Noticeably, many tunnel-like spaces are present between mesothelial cells. These empty spaces might serve as pathways for macrophages to extend their processes, resulting in apical macrophages having a dendritic shape. This speculation is consistent with the observation that apical macrophages can have a portion of their cell bodies and processes located beneath the mesothelial cell layer (see Figure 2).

In the middle and basal regions of the cochlea, mesothelial cells gradually shift from a bulky morphology to a flattened morphology, with the transition beginning at approximately 30–40% from the apex (Figure 5C). This transition site appears to correspond with the observed changes in the morphology of basilar membrane macrophages. Mesothelial cells in the middle and basal regions of the cochlea were scattered. The tunnel-like spaces between mesothelial cells were less abundant (Figure 5D) as compared with those observed in the apical section of the cochlea. Taken together, these SEM observations revealed clear architectural differences in the mesothelial cell layer along the cochlear spiral.

### 3.4. Distinct immune environments in the apical and basal regions of the cochlear spiral

To investigate biological factors that could contribute to the observed apical-to-basal difference in macrophage morphology, we examined the immune environment of the cochlea. We hypothesized that the apex-to-base differences in the morphology of basilar membrane macrophages might be related to differences in the immune microenvironment of these regions. To support this hypothesis, we compared mRNA expression of five immune-related genes (*Tnf*, *Il1*β, *Ccl2*, *Ccl3*, and *Ccl4*) between the apical and basal cochlear tissues collected from five young C57BL/6J mice of both sexes (4–6 weeks old, *n* = 5 biological replicates). We chose these immune molecules for analysis because they are known to be involved in cochlear inflammation (Patel et al., 2013; Yang et al., 2016).

**FIGURE 6:**
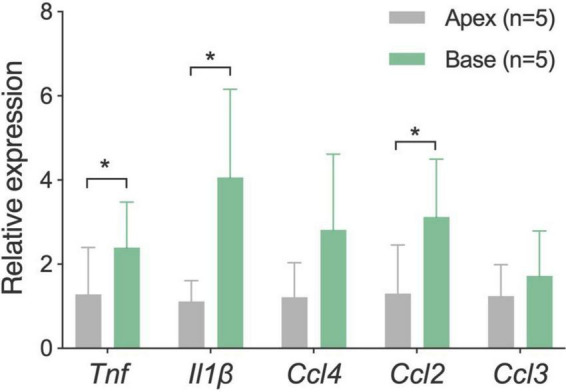
Comparison of mRNA expression levels of inflammatory genes between the apical and basal cochlear tissues. Tissues were separately harvested from the apical and basal regions of the cochlea (*n* = 5 biological replicates from five mice). Analysis revealed that three proinflammatory genes are expressed statistically at higher levels in the basal region of the cochlea as compared to the apical region. *P < 0.05; two-tailed paired Student’s *t*-test.

Our results revealed that three of the five examined immune-related genes displayed significant site-specific differences, with tissues from the basal portion of the cochlea exhibiting higher expression levels (*p* = 0.012 for *Tnf*, *p* = 0.04 for *Il1*β and *p* = 0.021 for *Ccl2*, two-tailed paired Student’s *t*-test) (Figure 6). It is worth noting that this difference was observed in normal cochleae, which provides evidence for the existence of intrinsic differences in immune microenvironments between the apical and basal regions of the cochlea. This difference may contribute to the observed differences in the morphologies of basilar membrane macrophages.

### 3.5. Morphological transformation of basilar membrane macrophages in aging cochleae

**FIGURE 7:**
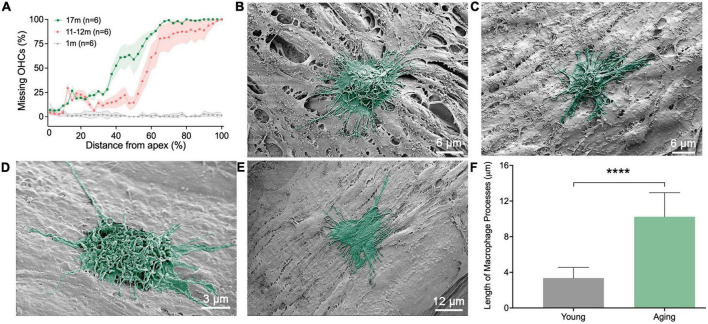
Scanning electronic microscopy (SEM) views of macrophages in aging ears. **(A)** Cochleogram showing the expansion of outer hair cell lesions with the increase of age. **(B,C)** Typical morphologies of macrophages on the apical region of the basilar membrane obtained from 11-month-old mice. Notice that the cells exhibit a rounded shape, characterized by numerous long and thin processes at their tops and along their edges. **(D,E)** Typical morphologies of macrophages in the middle and basal regions of the basilar membrane, obtained from 11-month-old mice. Like apical macrophages, the cells exhibit a rounded or irregular shape, characterized by numerous long and thin processes on top of the cell and along their edges. **(F)** Comparison of the length of macrophage surface processes between aging and young mice (*n* = 18 processes each group from 6 cochleae). ****P < 0.001; unpaired two-tailed Student’s *t*-test.

To provide additional evidence supporting the influence of the immune environment on macrophage morphology, we examined changes in macrophage morphology in aging ears. We collected nine cochleae from both male and female C57BL/6J mice aged between 11 and 19 months. Young mice aged 1–4 months were used as controls. Additionally, we conducted quantitative analysis of hair cells at three different time points (1, 11–12, and 17 months, *n* = 6 for each time point) to elucidate the progressive pathogenesis of sensory cells with increasing age (Figure 7A).

We noticed that, during the aging process, apical macrophages adopted certain morphological features of the middle and basal macrophages. Specifically, many macrophages altered their morphology from an elongated, dendritic shape seen in young cochleae to a more rounded shape commonly found in the middle and basal regions of the cochleae (Figures 7B, C). Furthermore, we found that some basal macrophages acquired certain features of macrophages found in the apical region of the cochlea. Notably, many macrophages developed multiple long, slender processes (Figures 7D, E), a trait commonly observed in apical macrophages. These thin, elongated processes intertwined on the macrophage surface. Statistical analysis of the length of the surface processes substantiated the difference between the young and aging macrophages (Figure 7F). In aging cochlea, the elongated processes of macrophages can improve their capacity to perform immunosurveillance over larger areas and reach cellular debris that is further away.

**FIGURE 8:**
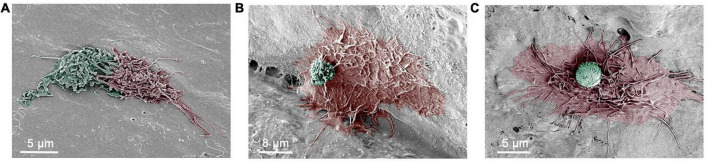
Scanning electronic microscopy (SEM) images of macrophage interactions. **(A)** An SEM image depicting the intermingling of surface processes of two macrophages in an 11-month-old mouse cochlea. **(B)** An SEM image showing a large macrophage with long and thin processes and a small round cell on its top. The small cell has microvilli and ruffles on its surface, which are morphological features of monocytes. The cochlea was collected from a 17-month-old mouse. **(C)** An SEM image showing a macrophage with long and thin processes along with a small round cell that exhibits a relatively smooth surface with small pits. The cochlea was collected from an 11-month-old mouse.

Another notable feature of macrophages in aging cochleae was an increase in the interaction between macrophages. In young cochleae, macrophages were scattered, and physical contact between the cells was rare. However, in aging ears, interactions between macrophages were frequently observed. These interactions occurred between cells with similar macrophage morphologies (Figure 8A) or between cells with different morphologies (Figures 8B, C). This cell-cell interactions were observed in a location-specific manner, primarily appearing in the middle and basal portions of the cochlear spiral.

## 4. Discussion

This study aimed to investigate the location-dependent morphology and functionality of cochlear macrophages bordering the scala tympani and identify potential factors that contribute to these macrophage properties. Our findings revealed significant differences in the topographical features and phagocytic activities of basilar membrane macrophages along the cochlear spiral, with the macrophages in the basal region exhibiting more activated phenotypes. We also demonstrated that there is a site-dependent variation in the expression levels of immune-related genes and the structural features of the basilar membrane mesothelial cell layer between the apical and basal regions of the cochlea, suggesting that different microenvironments exist for macrophages in these areas. Finally, our study showed that there is a switch of certain characteristics between apical and basal macrophages, with apical macrophages adopting some features of basal macrophages and basal macrophages acquiring certain traits similar to those of apical macrophages. Collectively, these findings suggest that, despite similar tissue composition, the apical and basal regions of the cochlea possess different structural and immune features.

Our long-term study goal is to elucidate the impact of cochlear macrophages on sensory cell homeostasis. Considering that macrophages exert stronger influences on neighboring cells in close proximity, we narrowed our focus to macrophages located in the immediate vicinity of outer hair cells. Cochlear macrophages reside in various regions of the cochlea, including the modiolus, ganglion cell regions, lateral wall, osseous spiral lamina, spiral limbus, and the luminal surface of the scala tympani and scala vestibuli. For our current study, we specifically focused on macrophages located on the basilar membrane due to their close proximity to the sensory cells in the organ of Corti. Moreover, these cells exhibit distinct differences in morphology between the apical and basal regions. Our previous studies have shown that basilar membrane macrophages alter their morphologies in response to sensory cell pathogenesis following acoustic overstimulation and age-related degeneration (Frye et al., 2017, 2018; Zhang et al., 2020). Thus, investigating macrophages on the basilar membrane offers valuable insights into the crosstalk between immune cells and sensory cells.

To explore the possible mechanisms responsible for shaping the location-dependent morphology, we examined the macrophage ecosystem. First, we observed the mesothelial cell layer because it provides structural support to macrophages on the basilar membrane. We found significant differences in the structural framework of the mesothelial cell layer along the cochlear spiral. In the apical portion, mesothelial cells are bulky, resulting in formation of numerous intracellular spaces. It appears that these intercellular spaces can provide avenues for macrophages to extend their processes toward the organ of Corti. Indeed, we observed many macrophages in this region with their cell bodies or processes located beneath mesothelial cells. In contrast, lack of such intercellular spaces in the middle and basal regions of the cochlea restricts macrophages from extending their processes to the organ of Corti. Thus, the physical structure of the mesothelial cell layer may influence macrophage morphology, particularly in the apical region of the cochlea.

Second, we compared the immune status between the apical and basal cochlear regions by examining the mRNA expression of immune-related genes, many of which possess pro-inflammatory functions. We found that tissues from the basal region of the cochlea displayed a higher expression level of pro-inflammatory genes. Considering that the cochleae used for this analysis were collected from mice at the age of 4–5 weeks, before the onset of age-related sensory cell pathogenesis, we posit that this intrinsic characteristic could potentially contribute to the early onset of basal sensory cell death observed in C57BL/6J mice (Johnson et al., 1997, 2017; Noben-Trauth et al., 2003). As macrophages can develop an amoeboid shape during proinflammatory activation (Buttini et al., 1996; Kloss et al., 2001; Frye et al., 2017), the pro-inflammatory environment in the basal region of cochlea may promote basal macrophages to adopt a rounded morphology. This speculation is supported by our current finding that, in aging cochleae when chronic inflammation occurs, apical macrophages transition from a branched shape to an amoeboid shape, which is a characteristic of basal macrophages. Thus, the distinct morphology of basilar membrane macrophages may reflect the difference in immune environments along the cochlear spiral.

How the apical-to-basal difference in morphology affects macrophage function remains unclear. Macrophages are known to have a range of morphologies that reflect their functional diversity. By analyzing the morphological characteristics of basilar membrane macrophages, it is possible to speculate that the dendritic morphology and elongated processes increase the contact areas of macrophages with surrounding tissues. Moreover, this morphology facilitates the protrusion of macrophages into the regions beneath mesothelial cells, enabling them to access the organ of Corti. Thus, this structural feature may suggest that apical macrophages provide better immune surveillance and protection to the organ of Corti compared to basal macrophages. The presence of this feature may explain why apical sensory cells are less susceptible to many pathological conditions. In contrast, macrophages in the middle and basal regions have a more rounded morphology and fewer pseudopodia compared to apical macrophages. This allows them to move quickly and efficiently over the mesothelial cell layer, which could be beneficial for macrophages in providing immune surveillance to the scala tympani space. Moreover, an amoeboid morphology enhances the cell’s ability to engulf large cell debris. Indeed, our *ex vivo* observation of phagocytic activities supports this speculation as basal macrophages are able to obtain more latex beads as compared to those in the apical region of the cochlea. Regarding the origins of cell debris that accumulates following cochlear damage, we speculate that these debris consist of deceased sensory cells, supporting cells, and macrophages. The current study used two stress conditions, aging and acoustic overstimulation. Both can induce sensory cell death as evidenced by the presence of sensory cell lesions reported in the current study for aging ears (Figure 7F) and our previous observations for acoustic injury (Yang et al., 2016; Zhang et al., 2020). Previous research has also indicated that the rupture of the reticular lamina results in the ejection of sensory cell debris, allowing it to enter the perilymph space (Bohne and Rabbitt, 1983; Thorne et al., 1984). In addition to sensory cell debris, dead macrophages could yield cellular debris. We previously observed a decrease in the number of macrophages during the recovery phase of acute acoustic injury (Yang et al., 2015). Local macrophage death may contribute to this reduction. Regardless of the origin of the cell debris, their removal is essential for promoting the recovery process in the damaged cochlea.

The SEM technique is not a new method. It has been utilized for observing cochlear tissues for many years (Lim and Lane, 1969; Lim, 1970; Lim and Melnick, 1971). However, based on our literature review, there have been no previous reports of SEM analysis of cochlear macrophages, except for the observations from our lab (Hu et al., 2018; Zhang et al., 2020). The lack of prior observations of cochlear immune cells using SEM could be attributed to the limitations of early SEM techniques, including inadequate resolution, sensitivity, sample preparation, or other technical factors that prevented clear observation of cochlear immune cells. After examining previously published SEM images, we discovered that certain structures on the basilar membrane, osseous spiral lamina, and lateral wall exhibited macrophage features but were misidentified as cell nuclei or basilar membrane cells, likely due to the lack of structural details presented in these images. Thus, our current study represents a significant improvement in SEM technology and sample preparation. By utilizing newer SEM technology and improved tissue preparation techniques, we were able to clearly identify and characterize macrophages in the cochlea. Our findings not only enhance our understanding of the role of immune cells in the cochlea but also demonstrate the potential of SEM as a valuable tool for future studies in this area.

## Data availability statement

The raw data supporting the conclusions of this article will be made available by the authors, without undue reservation.

## Ethics statement

The animal study was reviewed and approved by the Institutional Animal Care and Use Committee of the State University of New York at Buffalo.

## Author contributions

BH and CZ contributed to the conception, design, methodology, execution of the study, and wrote the draft of the manuscript. CZ and MY contributed to data analyses and figures creation. BH contributed to project administration and supervision. PB contributed to SEM. All authors contributed to the manuscript revision, read, and approved the submitted version.
